# Exploring the Impact of Unsafe Behaviors on Building Construction Accidents Using a Bayesian Network

**DOI:** 10.3390/ijerph17010221

**Published:** 2019-12-27

**Authors:** Shengyu Guo, Jiali He, Jichao Li, Bing Tang

**Affiliations:** 1 School of Economics and Management and Institute of Management Science and Engineering, China University of Geosciences, Wuhan 430000, China; guoshy@cug.edu.cn (S.G.); hejiali0217@csu.edu.cn (J.H.); tangbing@cug.edu.cn (B.T.); 2 Business School, Central South University, Changsha 410000, China

**Keywords:** chains of unsafe behaviors, Bayesian network, building construction, accident prevention, probabilistic transmission path

## Abstract

Unsafe behavior is a critical factor leading to construction accidents. Despite numerous studies supporting this viewpoint, the process by which accidents are influenced by construction workers’ unsafe behaviors and the extent to which unsafe behaviors are involved in this process remain poorly discussed. Therefore, this paper selects cases from Chinese building construction accidents to explore the probabilistic transmission paths from unsafe behaviors to accidents using a Bayesian network. First, a list of unsafe behaviors is constructed based on safety standards and operating procedures. Second, several chains of unsafe behaviors are extracted from 287 accident cases within four types (fall, collapse, struck-by and lifting) to form a Bayesian network model. Finally, two accidents are specifically analyzed to verify the rationality of the proposed model through forward reasoning. Additionally, critical groups of unsafe behaviors leading to the four types of accidents are identified through backward reasoning. The results show the following: (i) The time sequence of unsafe behaviors in a chain does not affect the final posterior probability of an accident, but the accident attribute strength of an unsafe behavior, affects the growth rate of the posterior probability of an accident. (ii) The four critical groups of unsafe behaviors leading to fall, collapse, struck-by, and lifting are identified. This study is of theoretical and practical significance for on-site behavioral management and accident prevention.

## 1. Introduction

The construction industry is characterized by intensive labor, congested worksites and a tough environment, and it is thus regarded as one of the most high-risk industries worldwide [1,2]. As the statistics show, fatal occupational injuries during goods production numbered 1967 in the United States in 2017, with construction employees representing 49.4% [3]. Fatal injuries in the main industries numbered 147 in Great Britain in 2018, and construction workers represented 20.4% [4]. The statistics showed that the number of laborer deaths was 909 in Japan in 2018, and construction workers accounted for 34.7% [5]. Compared with these developed countries, the number of accidents and fatal injuries are even higher in the Chinese construction industry due its short history and rapid development. As shown on the Ministry of Emergency Management of the People’s Republic of China website [6], over 13,000 construction workers died from 2014–2018, with an average of 35 fatalities each day. These statistical data reflect that accident prevention remains a serious task in the construction industry.

In accident causation theories, Heinrich et al. [7] found that unsafe behavior by humans was the main cause of accidents based on numerous statistical data. Salminen and Tallberg [8] noted that 84–94% of accidents were due to human errors after studying 178 fatal occupational injuries and 99 major casualties. Fang et al. [9] noted that unsafe behavior by workers is the most frequent and direct cause of on-site accidents. Therefore, unsafe behavior is a critical factor affecting the occurrence of accidents and should be prevented. For accident prevention, existing studies have mainly discussed the correlations between unsafe behaviors and various factors such as unsafe conditions [10], and inadequate management and organization [11,12], but they have rarely discussed the process and extent of unsafe behaviors causing accidents. In fact, an accident often involves multiple unsafe behaviors. These unsafe behaviors have time sequences (objectively occurring in chronological order) and thus form an accident chain, that is, a chain reflects the process of multiple unsafe behaviors causing an accident. Bayesian networks (BNs) are a powerful way of handling uncertainty, especially when there are causal relationships between various events [13]. By integrating multiple chains into a BN, the probabilistic transmission paths from unsafe behaviors to accidents can be analyzed. Therefore, causal relationships are built between unsafe behaviors and accidents to reflect the impact of each unsafe behavior on different types of accidents. In brief, the process and extent of unsafe behaviors causing accidents can be understood in depth by using a BN, which is of theoretical and practical significance for on-site behavioral management and accident prevention in the construction industry.

This paper uses a case study approach, selecting building construction accidents in China for the analysis. The chains of unsafe behaviors from historical accident cases are extracted and further used to construct a Bayesian network model of building construction accidents (BNMBCAs). Then, BNMBCAs are analyzed through forward and backward reasoning to explore the process and extent of unsafe behaviors causing accidents.

### 1.1. Correlations Between Unsafe Behaviors and Various Factors in the Construction Industry

Since Heinrich revealed the association underpinning unsafe behavior and work-related accidents, unsafe behavior at work has drawn the attention of researchers from different areas. Likewise, as a typical high-risk trade, construction workers’ unsafe behavior has been studied extensively from the organizational level to the individual level [14]. For example, the organizational factors such as the safety climate [15] and group norms [16] were identified as the important contributors to the workers’ unsafe behavior, and also the influence of critical managerial roles in an organization, such as the supervisors, was also highlighted in multiple studies [17,18]. Some researchers devoted their efforts into investigating the frontline workers’ behavior from various perspectives, including the workers’ physical effects [19], psychological conditions [20,21] and cognitive process [22,23]. However, on the other hand, compared with the fact that the unsafe-behavior is profiled from various perspectives at different levels, another core part of the fundamental Heinrich theory, namely, the statistical association between unsafe behavior and accidents was somehow ignored [10,24]. In other words, the impact of different unsafe behaviors on the occurrence of accidents remains poorly discussed from a quantitative point of view. Furthermore, such a research gap also results in the difficulties of implementing the behavioral-based measures into a safety management practice on site [25].

### 1.2. Applications of a Bayesian Network in Occupational Safety

Existing studies have applied BNs to safety assessments in several high-risk locations and tasks, such as oil and gas pipelines [26], natural gas stations [27], maritime work [28], hazmat transportation [29], and construction. Chen and Leu [30] assessed the probabilities of fall risks and their sensitive factors in bridge projects based on BN analysis. Wu et al. [31] proposed a dynamic BN-based approach to provide support for safety analysis in tunnel construction. Wang and Chen [32] established a fuzzy comprehensive BN for safety risk assessment at metro construction projects. Xia et al. [33] used a BN model to quantify the total influences of risk factors at five distinct levels for predicting safety performance in construction projects. For unsafe behavior analysis, Mohammadfam et al. [34] constructed a BN model and determined the three best predictors of employees’ safety behavior at workplaces. Ghasemi et al. [35] identified critical factors affecting high-risk unsafe behaviors at workplaces based on BN analyses. Aliabadi et al. [36] found contributing factors to unsafe acts in mining accidents through the BN. Jitwasinkul et al. [12] used a BN to explore the influence of organizational factors and their impact on safe work behavior in the Thai construction industry. In brief, BNs are effective for identifying critical factors affecting unsafe behaviors for accident prevention. However, this approach has not been used to analyze the influence probability and the transmission path of unsafe behaviors on the accident.

## 2. Methodology

To analyze the process and extent of unsafe behaviors causing accidents in construction, a case study approach was adopted [37]. Building construction is a typical high-risk construction type. Aneziris et al. [1] indicated that the building construction industry greatly contributes to the overall number of occupational accidents. Cheng et al. [38] noted that 57% of 800 occupational accident records were related to building construction projects. This situation is more serious in China. Li et al. [39] showed that China accounted for 53% of the world’s 200 m-plus buildings in 2017. Therefore, accident cases from the Chinese building construction industry were selected for analysis.

### 2.1. Data Collection and Classification

Before extracting chains of unsafe behaviors from accident cases, these unsafe behaviors need to be defined. Combining with the relevant safety standards and operating procedures, a list of unsafe behaviors was initially formed. The unsafe behaviors on this list are mainly extracted from the *Classification Standard for Casualty Accidents of Enterprise Workers* (GB 6441-1986), which includes 49 unsafe behaviors. Other guidelines for safety standards and operating procedures in China, such as the *Technical Code for Safety of Working at Height of Building Construction* (JGJ 80-2016) and *Code for Construction and Acceptance of Crane Installation Engineering* (GB 50278-2010), are also used as references to list unsafe behaviors. From these data sources, a total of 73 unsafe behaviors across 19 types of building construction were identified.

**Figure 1:**
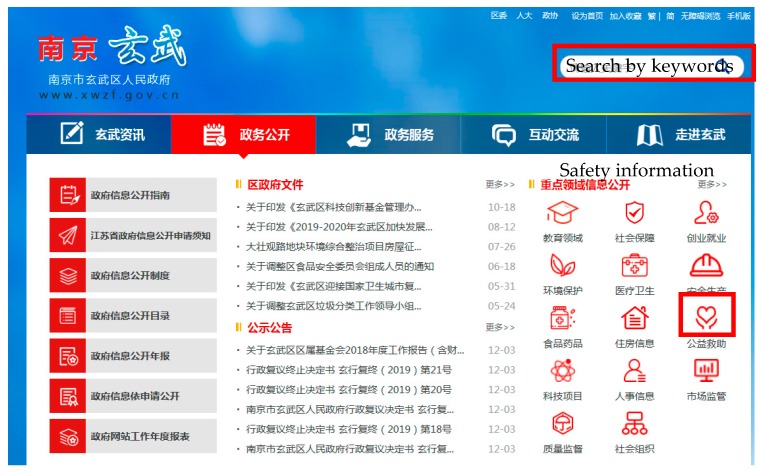
The interface of the government website to illustrate the searching process.

Accident cases were then collected from government websites in China, mainly from the Ministry of Housing and Rural-Urban Development and the Work Safety Administration in selected provinces between 2010 and 2018. All the collected accident cases are fatal accidents. Non-fatal accidents were investigated by related construction companies, but the reports were not released on websites, so little information could be used to extract chains. One example of the searching process is shown in Figure 1. The detailed reports of accident cases are found in the safety information section of the government website. Furthermore, accident cases can be directly searched by keywords such as construction accident, accident report and accident investigation. Through the process, a total of 303 accident cases were initially collected, in which four accident types including fall (F), collapse (C), struck-by (S) and lifting (L) accounted for more than 94%. The amount of data was affected by three factors: (i) Workers’ unsafe behaviors should be contained in the selected accidents. Some accidents caused by unsafe conditions or the environment were not selected. (ii) The selected accidents should have detailed reports. Many accidents were only recorded with a simple process, and thus chains of unsafe behaviors could not be extracted. (iii) Many accident reports are only released for a period of time, and then are removed from government websites. However, some representative accidents can still be found. Although there was not much data, all accident cases involved workers’ unsafe behaviors and had detailed reports. Additionally, many representative accidents of building construction in China were included. In terms of the collected accident cases, there were too few other types of accidents such as electrical shock, and thus they were not considered for analysis. Ultimately, 287 accident cases were identified, of which F has the largest number (191 cases), followed by C (39 cases), S (33 cases), and L (24 cases).

### 2.2. Bayesian Network

BNs have been widely used in many research fields since they were first proposed by Pearl [40]. A BN is effective for uncertain knowledge expression and reasoning; thus, it is suitable for exploring the probabilistic transmission paths from unsafe behavior to accidents. A BN is composed of two parts: a directed acyclic graph (DAG) and an associated joint probability distribution (JPD) [31]. The DAG of the BN model is composed of variables and directed arrows between variables. The JPD of the BN model mainly refers to the conditional probability table (CPT) set of a BN. In a BN, the root nodes are those nodes with no arrows pointing to them, and they have corresponding prior probability distributions. The node at which the arrow starts is the parent node, and the node the arrow points to is called the child node. Each node has a CPT, which is used to represent the relationship between the node and its parent node [32].

IBM SPSS Modeler 14.2 (IBM, Arnmonk, NY, USA) and Netica 5.18 (Norsys Software Corp, Vancouver, BC, Canada) were selected to construct the BN model for analysis and reasoning, respectively. IBM SPSS Modeler is a widely used software package for data mining, in which the Bayesian modeling function has advantages in parameter learning. In addition, the interface is simple, and the graphs generated by the BN have a good visualization effect. Netica is another widely used BN software package that has strong reasoning ability for risk prediction and cause diagnosis of accidents. Therefore, IBM SPSS Modeler 14.2 was used to construct a BNMBCA to generate the network topology and its corresponding CPT with different unsafe behaviors as the root nodes. Then, the constructed model was rebuilt in Netica 5.18 for forward and backward reasoning.

The BN forward reasoning can predict the probability of the occurrence of risk events and thus take preventive measures early, when the cumulative effects of contributing factors are large. It is assumed that the set of all unsafe behaviors with known risk states is the evidence *B_c_* during the construction process (*B_c_* is an empty set when there is no evidence), and the probability of occurrence of an accident is *Y*. The formula for forward reasoning is as follows:

(1) $$P\left(Y_{k}\left|B_{c}\right.\right)=P\left(Y_{k}\left|B_{1},B_{2},\cdots ,B_{i}\right.\right)=\frac{P\left(y_{i},B_{1},B_{2},\cdots ,B_{i}\right)}{P\left(B_{1},B_{2},\cdots ,B_{i}\right)}$$

where *Y_k_* ∈ {S, C, F, L}, *P*(*B_i_*) = 1 when the risk state of the unsafe behavior *B_i_*, is known. The BN backward reasoning aims to find the cause of known results and calculate its posterior probability, which is often used in pathological diagnosis [41] and fault diagnosis [42]. Assuming an accident *Y_k_* occurs, then the posterior probability of the first unsafe behavior *B_i_* is calculated as follows:

(2) $$P\left(\left.B_{i}=yes\right|\right.\left.Y_{k}=yes\right)=\frac{P\left(B_{i}=yes\right)P\left(\left.Y_{k}=yes\right|\right.\left.B_{i}=yes\right)}{P\left(Y_{k}=yes\right)}$$

The larger *P*(*B_i_* = *yes*|*Y_k_* = *yes*) is, the greater the impact of the unsafe behavior *B_i_* is in a certain accident type. The followed unsafe behaviors are continuously diagnosed through backward reasoning to identify the critical groups of unsafe behaviors leading to the four accident types.

## 3. Process and Result

### 3.1. Extracting Unsafe Behaviors

According to the time sequences of unsafe behaviors in the accidents, 211 chains composed of 37 unsafe behaviors from the list were extracted from the four types of accidents. The accidents caused by single unsafe behaviors were eliminated from analysis; and the nodes of the 37 remaining unsafe behaviors are seen in the Appendix A (there are 40 unsafe behaviors occurring in all types of accidents, but 37 in the four types of accidents). The chains of unsafe behaviors are directed chains, in which the nodes represent codes of unsafe behaviors and the arrow directions indicate the time sequences of unsafe behaviors. For instance, the extraction of a chain of unsafe behaviors in a fall accident is shown in Table 1.

### 3.2. Constructing Bayesian Network

**Figure 2:**
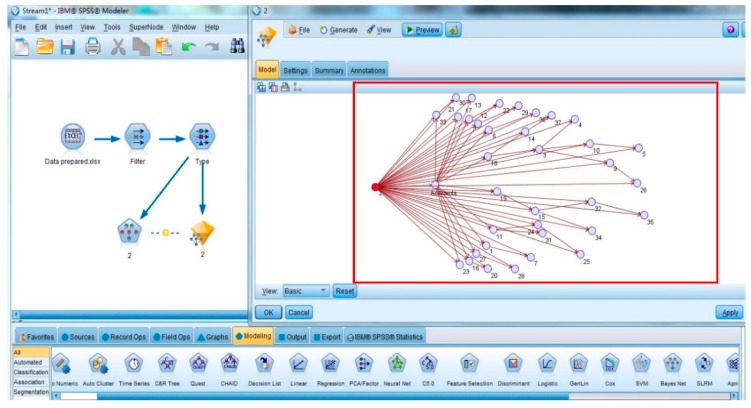
The structure of the BNMBCA with *B*_2_ as the root node.

After the chains of unsafe behaviors were extracted, the BNMBCA was constructed by IBM SPSS Modeler 14.2. First, the input file was created by Microsoft Excel 2007 (Microsoft, Redmond, WA, USA), which comprises 287 rows (accident cases) and 37 columns (unsafe behaviors). In each row, any unsafe behavior that occurs in an accident is marked as “Yes”; otherwise, it is marked as “No”. Second, the structural and parameter learning method of BNMBCA was designed. There are two types of BN structures in the software package, tree augmented native (TAN) and Markov blanket. In addition, parameter learning methods also have two options: maximum likelihood and Bayesian adjustment for small cell counts. The contributions of all unsafe behaviors to accidents can be considered in a TAN. Moreover, Bayesian adjustment for small cell counts can modify the prior probabilities of unsafe behaviors with fewer occurrences and an uneven distribution in the original data sample to obtain more reasonable results. Therefore, the TAN was selected as the network structure, and the Bayesian adjustment for small cell count was selected as the parameter learning method. Finally, the file was input into the software package, and root nodes were identified to generate different structures of BNMBCA. Taking *B*_2_ as the root node as an example, the visualization of BNMBCA is shown in Figure 2. In addition, the CPT of the four types of accidents is shown in Table 2. The structures of the BNMBCA and the CPTs are variable with different root nodes. However, the JPD of any nodes is consistent regardless of which node is selected as the root node.

### 3.3. Forward and Backward Reasoning of the BNMBCA

Before the forward and backward reasoning of the BNMBCA, an unsafe behavior node should be identified as the root node. The root node should meet two requirements: (i) The identified unsafe behavior occurs frequently in the four types of accidents, and (ii) the identified unsafe behavior contributes to the occurrence of the four types of accidents. The contribution of an unsafe behavior to the occurrence and development of a certain type of accident is defined as the accident attribute of the unsafe behavior, and the strength of the accident attribute reflects its degree of contribution. Therefore, an unsafe behavior node with a weak accident attribute was selected as the root node. Based on the statistics, the occurrences of several unsafe behaviors in the four types of accidents are shown in Table 3. It can be seen that *B*_14_ (engage in specialized operation without a permit) occurs frequently and contributes to the four types of accidents. Thus, *B*_14_ is suitable for selection as the root node, and then the structure of the BNMBCA built by IBM SPSS Modeler 14.2 is rebuilt by Netica 5.18 for forward and backward reasoning.

#### 3.3.1. Forward Reasoning for Verifying the Rationality of the BNMBCA

**Figure 3:**
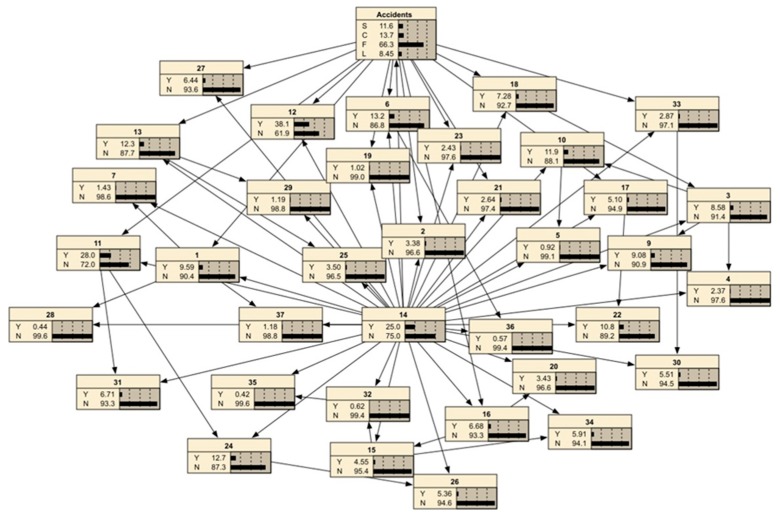
Bayesian network with no evidence.

**Figure 4:**
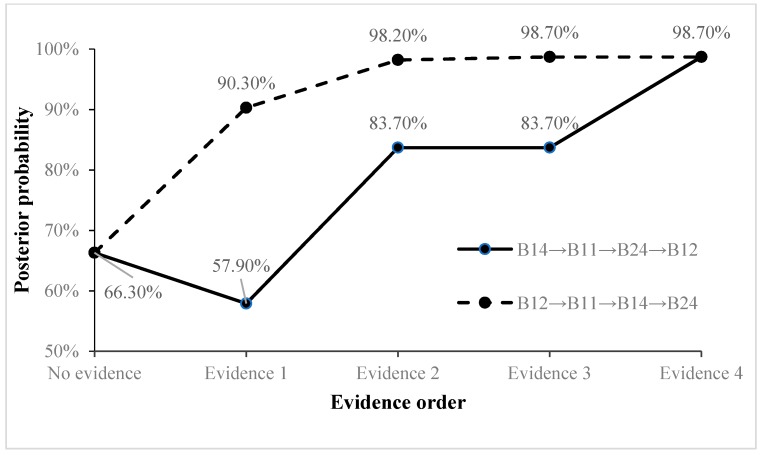
The change in the trend of the posterior probabilities of F.

When certain unsafe behaviors occur at a building construction site, the probability of the four types of accidents can be predicted by the forward reasoning of the BNMBCA. However, the rationality of the BNMBCA should be verified first. Using the collected accident cases, predictions without and with evidence are compared to prove the rationality of the BNMBCA. Additionally, the impact of multiple unsafe behaviors on accidents is further explored. Two accidents were selected for analysis; the first is a fall accident, and the chain of unsafe behaviors is “*B*_14_→*B*_11_→*B*_24_→*B*_12_”. With no evidence prediction, the prior probability of root node *B*_14_ (engage in specialized operation without a permit) is identified as 25% according to the rate of this unsafe behavior in contributing to the four types of accidents. Then, the posterior probabilities of the accidents are 11.6% (S), 13.7% (C), 66.3% (F), and 8.4% (L). The visualization of the BNMBCA is shown in Figure 3. With evidence prediction, *B*_14_ is initially inputted as the evidence node, the posterior probability of F decreases to 57.9% and the posterior probabilities of S, C and L slightly increase. Then, *B*_11_ (implement inadequate protection measures for holes or borders), *B*_24_ (do not use up safety net as required), and *B*_12_ (do not use all personal protective equipment (PPE)) are input in order as evidence nodes, and the posterior probability of F sharply increases to 98.7%. The result indicates that falling is most likely to occur when these unsafe behaviors occur. By changing the input order of these evidence nodes, the growth rate of F changes. That is, the accident attributes of these unsafe behaviors are different. The posterior probability of F greatly increases when *B*_11_ and *B*_12_ are evidence nodes, so these two unsafe behaviors have strong accident attributes, while *B*_14_ and *B*_27_ have weak accident attributes. However, the final posterior probability of F is still 98.7%, and this does not change with the change in input order for the evidence nodes. Figure 4 reflects the change in the trend of the posterior probabilities of F when the input orders of the evidence nodes are “*B*_14_→*B*_11_→*B*_24_→*B*_12_” and “*B*_12_→*B*_11_→*B*_14_→*B*_24_”, respectively.

The second is a lifting accident, and the chain of unsafe behaviors is “*B*_14_→*B*_18_→*B*_27_→*B*_6_”. Predictions without and with evidence were carried out, and the posterior probabilities of the four types of accidents are shown in Table 4. When *B*_14_ (engage in specialized operation without a permit), *B*_18_ (install or dismantle machines and equipment against procedures), *B*_27_ (issue improper commands) and *B*_6_ (operate machines and equipment against the guidelines) all occur, the posterior probability of L increases to 87.2%. Moreover, the posterior probabilities of L increase greatly with the occurrence of *B*_18_ and *B*_27_, which have strong accident attributes.

Based on the analysis of the above two accidents, the findings are as follows: (i) Compared with the no evidence prediction, the posterior probabilities of accidents are significantly higher under prediction with evidence. Moreover, the posterior probabilities of accidents continuously increase with the occurrence of unsafe behaviors in chains. Therefore, the rationality of BNMBCA is verified. The BNMBCA can be used to predict the four types of accidents that may occur given multiple unsafe behaviors. (ii) The sequence of unsafe behaviors does not affect the final posterior probabilities of the accidents, but it affects the growth rate of the posterior probabilities of the accidents. That is, the strength of the accident attribute of unsafe behaviors promotes the growth rate of accident probabilities. In brief, the unsafe behaviors in the two accident cases are inputted into the BNMBCA as evidence nodes, and the final posterior probabilities are *P*(F) = 98.7% and *P*(L) = 87.2%, respectively. The findings indicate that BNMBCA can be used to predict the four types of accidents after multiple unsafe behaviors occur, and the backward reasoning of the BNMBCA can be further constructed.

#### 3.3.2. Backward Reasoning for Identifying Critical Unsafe Behaviors

The forward reasoning of the BNMBCA verifies the rationality of the proposed model; thus, the BNMBCA can be further applied to backward reasoning for risk diagnosis. In the process of risk diagnosis, the posterior probability is a critical index for analyzing the impact of multiple unsafe behaviors on accidents. When a certain type of accident is analyzed, the posterior probability of each unsafe behavior is deduced by adjusting the accident probability in the BNMBCA. Generally, the unsafe behavior with the largest posterior probability represents the critical unsafe behavior leading to a certain type of accident. Then, this unsafe behavior is inputted as a new evidence node and the posterior probabilities of related unsafe behaviors in the chains are updated to find the next critical unsafe behavior. When the posterior probabilities of the remaining unsafe behaviors are below a certain value indicating that these unsafe behaviors are less likely to occur, the diagnostic process is completed. Through this process, the four types of accidents were diagnosed to explore groups of unsafe behaviors that need to be critically controlled in daily construction activities.

**Figure 5:**
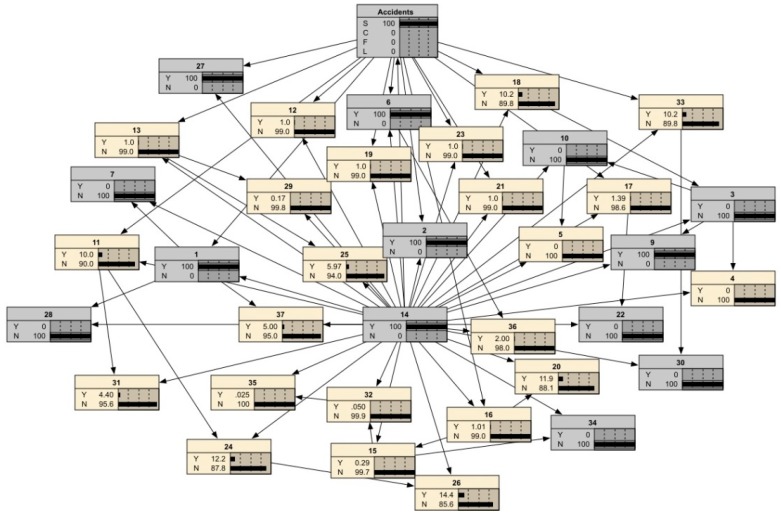
The diagnosis result for the S accident type.

Taking the S accident type as an example, the accident probability is adjusted to *P*(A) = 100%, and the largest posterior probability is *P*(*B*_14_ = Y) = 33.3% after probability updating, so *B*_14_ is identified as a critical unsafe behavior. To prevent this type of accident, increased attention should be given to checking whether specialized operators have received professional training and obtained permits. Then, *P*(*B*_14_ = *Y*) = 100% is adjusted to continue the diagnosis process. Considering the occurrence of unsafe behaviors in the S accident type among the 303 accident cases, unsafe behaviors with a frequency higher than 8% are identified as potentially happening, and otherwise as not happening. When *P*(*B*_14_ = Y) = 100% is input, the largest posterior probability is *P*(*B*_6_ = *Y*) = 46.0%. Additionally, the occurrence of this unsafe behavior is higher than 8%, and so it is determined to be the second critical unsafe behavior. During building construction, potential unsafe behaviors of machine operators need to be addressed to prevent the S type accidents. The diagnosis process is completed when the posterior probabilities of all the remaining unsafe behaviors are less than 15%. As shown in Figure 5, the critical group of unsafe behaviors is identified as {*B*_14_, *B*_6_, *B*_9_, *B*_1_, *B*_2_, *B*_27_}.

The other three types of accidents were analyzed according to the diagnosis process. Therefore, the critical group of unsafe behaviors of C is {*B*_17_, *B*_14_, *B*_22_}, the critical group of unsafe behaviors of F is {*B*_12_, *B*_11_, *B*_24_, *B*_14_, *B*_31_, *B*_9_, *B*_13_}, and the critical group of unsafe behaviors of L is {*B*_6_, *B*_14_, *B*_18_, *B*_10_, *B*_3_, *B*_9_, *B*_27_, *B*_12_}. Compared with other combinations of unsafe behaviors, these four critical groups of unsafe behaviors reflect unsafe behaviors that have a greater impact on the four types of accidents and thus should be collaboratively controlled in building construction. Furthermore, as shown in Table 5, corrective measures are suggested for the unsafe behaviors in these groups, including safety inspection, safety training for workers and safety training for managers/engineers based on the previous work [43,44]. For the unsafe behaviors, such as engaging in a specialized operation without a permit, which will not cause accidents immediately, safety inspection should be strictly executed before and during construction tasks. The total number of migrant workers is over 50 million in 2018 [45], many workers have not received enough safety training to enhance their safety awareness. Therefore, safety training such as the use of PPE should be conducted for workers to prevent accidents and injuries. For the unsafe behavior *B*_27_ (issue improper commands), safety training should be developed for managers/engineers to improve their professional skills for struck-by and lifting accident prevention.

## 4. Discussion

Construction is one of the most hazardous industries; there are frequent accidents, and unsafe behavior is a critical factor. To explore the influence of unsafe behaviors and their impacts on accidents, a case study in the Chinese building construction industry was conducted to analyze the probabilistic transmission paths from unsafe behaviors to accidents using a BN. The data prepared for analysis included 287 accident cases of four types (F, C, S and L) extracted from government websites, and a list of 73 unsafe behaviors across 19 types was used to identify the unsafe behaviors in these cases. Then, the BNMBCA was constructed by 211 chains composed of 37 unsafe behaviors. The rationality of the BNMBCA was verified through forward reasoning, and four critical groups of unsafe behaviors leading to certain types of accidents were identified through backward reasoning. The contributions of this research are twofold as follows.

The unsafe behaviors contributed to the occurrence and development of construction accidents to varying degrees. In the process of forward reasoning, it was found that the time sequences of unsafe behaviors did not affect the final posterior probability of accidents, but the contributions of the unsafe behaviors to the increase in accident probability were different. The contributions of the unsafe behaviors depended on the complexity and strength of their accident attributes. If the accident attribute of an unsafe behavior is single and strong, the probability of the related accident will be greatly increased. Therefore, the unsafe behaviors that greatly contribute to certain types of accidents should be critically controlled. For instance, workers should implement protection measures for holes/borders, and the correct use of PPE will effectively prevent falling accidents. Chi et al. [46] also pointed out that workers’ unsafe behaviors had operation-specific characteristics and thus could be closely related to accident types. However, their study focused on the impact of different risk combinations of working conditions and workers’ behaviors while our study explored the different impacts of specific unsafe behaviors on accident types.

Four groups of unsafe behaviors, with the greatest impact on the four types of accidents, were identified. The four groups of unsafe behaviors derived from the backward reasoning represent the groups of unsafe behaviors that most likely lead to the four types of accidents, which were also identified as critical factors in several existing studies [30,47,48]. Thus, the unsafe behaviors in these groups should be collaboratively controlled during construction. Additionally, these critical groups of unsafe behaviors were identified based on reasoning from the probabilistic relations among several unsafe behaviors. That is, the co-occurrence of unsafe behaviors in these groups has a great impact on accidents. Therefore, managers/engineers should exert timely control when they find unsafe behaviors in these groups routinely occurring on site to avoid the diffusion effect of unsafe behaviors leading to accidents. For instance, managers/engineers should stop the construction tasks of machine operators to prevent struck-by accidents when machine operators are found to be working without permits and operating against procedures. Additionally, different corrective measures for controlling the unsafe behaviors in these groups could be taken to prevent accidents, including safety inspection, safety training for workers and safety training for managers/engineers. Moreover, these critical groups of unsafe behaviors could provide support for reason diagnosis during accident investigation.

Some limitations should be addressed in the data and model of this research. The number of accident cases selected was insufficient to obtain further findings due to the lack of detailed reports on accident causes and processes on government websites. SPSS Modeler constructed the Bayesian model with non-informative priors, so the posterior probability of an unsafe behavior node cannot be adjusted by its prior probability identified from historical accident data. In regard to these contributions and limitations, further research will be required to collect accident cases through more methods, such as interviews with companies engaged in building and other types of construction. Furthermore, MATLAB (MathWorks, Natick, Massachusetts, USA) can be considered for constructing the Bayesian model for inputting prior probabilities of unsafe behavior nodes.

## 5. Conclusions

Unsafe behaviors are regarded as the main factor causing accidents, but few studies have discussed the impact of multiple unsafe behaviors within accidents. This paper used the Chinese building construction industry as a case study to analyze the probabilistic transmission paths from unsafe behaviors to accidents using a BN. Based on the forward reasoning, the findings reflect that unsafe behaviors have different contributions in the occurrence and development of accidents. Additionally, critical groups of unsafe behaviors were identified for the prevention of certain types of accidents through the backward reasoning. From a theoretical perspective, accident causation theory has been improved through this work. Construction accidents were found to commonly be caused by multiple unsafe behaviors. The proposed model built probability connections between unsafe behaviors in accidents using a BN to explain the impact of unsafe behaviors on accidents depending on their accident attributes. Moreover, critical groups of unsafe behaviors were identified to emphasize the superposition effect of certain unsafe behaviors, which has a great impact on certain types of accidents. These findings help one to understand the different impacts of different unsafe behaviors on accidents, to improve accident causation theories. From a practical perspective, more detailed information can be provided to control unsafe behaviors by workers. The risk levels of unsafe behaviors can be identified in certain types of accidents by considering their accident attributes. Once managers/engineers know that the same unsafe behavior has different influences on different types of accidents, they can develop control strategies at locations with a high-frequency of different types of accidents on site. Furthermore, critical unsafe behaviors in the groups should be collaboratively controlled to cut the connection between the co-occurrence of unsafe behaviors to reduce the probability of potential accidents and incidents. The flexibility and applicability of the findings on the impact of unsafe behaviors in accidents should be further proven through comparative analysis among construction types (e.g., railway, road and building) in China and around the world.

## Figures and Tables

**Table 1:** One example of extracting a chain of unsafe behaviors in a fall accident.

Accident Type	Fall
Occurrence Process	Two workers installed air-conditioning condensing pipes on the exterior wall of a building. The bracket of their hanging basket was fixed on the roof of the twenty-first floor, and the wire rope of the hanging basket on the west side was fixed on the bracket. However, the wire rope on the east side was fixed on the vertical structure of the glass curtain wall instead of on the bracket. Neither of the workers wore safety belts during the operation. When they installed the air-conditioning condensing pipe on the eighth floor, the wire rope on the east side was loose causing the east side of the basket to be imbalanced. The two workers fell 30 m to the ground.
Accident Causes	1. The workers fixed the wire rope on the east side to the vertical structure of the glass curtain wall instead of the bracket. 2. The workers engaged in high-altitude operations without a permit. 3. The workers did not wear safety belts during the operation.
Time Sequence of Unsafe Behaviors	Install or dismantle machines and equipment against procedures→engage in specialized operation without a permit→does not use all personal protective equipment (PPE)
Chain	*B_1_*_8_→*B*_14_→*B*_12_

**Table 2:** The CPT of the four types of accidents when *B*_2_ is identified as the root node.

Root Node	CPT			
*B*_2_	S	C	F	L
Yes	0.43	0.07	0.43	0.17
No	0.10	0.14	0.68	0.09

**Table 3:** The occurrences of several unsafe behaviors in the four types of accidents.

Accident Type	Unsafe Behavior Node										
	*B*_12_	*B*_11_	*B*_14_	*B*_6_	*B*_24_	*B*_13_	*B*_1_	*B*_31_	*B*_9_	*B*_16_	*B*_18_
F	99	73	42	13	30	32	14	22	16	11	10
C	5	2	8	2	2	2	0	2	0	9	0
S	1	4	11	10	3	0	9	3	4	3	2
L	4	1	11	12	0	0	5	1	3	0	9
All	109	80	72	37	35	34	28	28	23	23	21

**Table 4:** Prediction without and with evidence for the second accident.

Evidence	*P*(S)	*P*(C)	*P*(F)	*P*(L)
No	11.60%	13.70%	66.30%	8.45%
*P*(*B*_14_) = 1	15.40%	11.30%	57.90%	15.40%
*P*(*B*_14_) = 1, *P*(*B*_18_) = 1	8.37%	1.23%	37.70%	52.70%
*P*(*B*_14_) = 1, *P*(*B*_18_) = 1, *P*(*B*_27_) = 1	8.71%	0.13%	10.30%	80.80%
*P*(*B*_14_) = 1, *P*(*B*_18_) = 1, *P*(*B*_27_) = 1, *P*(*B*_6_) = 1	9.39%	0.02%	3.39%	87.20%

**Table 5:** Corrective measures for the unsafe behaviors in the four groups.

Accident Type	Unsafe Behaviors	Corrective Measures
S	Engage in specialized operation without a permit	Safety inspection
	Operate machines and equipment against the procedures	Safety training for workers
	Use machines and equipment without conforming safety before	Safety inspection
	Stay in unsafe areas	Safety inspection
	Enter into dangerous areas	Safety training for workers
	Issue improper commands	Safety training for managers/engineers
C	Install or dismantle formwork support system against procedures	Safety training for workers
	Engage in specialized operation without a permit	Safety inspection
	Concreting against procedures	Safety inspection
F	Does not use all personal protective equipment (PPE)	Safety training for workers
	Implement inadequate protection measures for holes or borders	Safety inspection
	Do not set up safety nets as required	Safety inspection
	Engage in specialized operation without a permit	Safety inspection
	Do not hang warning signs in dangerous areas	Safety inspection
	Use machines and equipment without conforming safety before	Safety inspection
	Misuse PPE	Safety training for workers
L	Operate machines and equipment against the procedures	Safety training for workers
	Engage in specialized operation without a permit	Safety inspection
	Install or dismantle machines and equipment against procedures	Safety training for workers
	Use machines and equipment without effective safety devices	Safety inspection
	Use overloaded machines	Safety inspection
	Use machines and equipment without conforming safety before	Safety inspection
	Issue improper commands	Safety training for managers/engineers
	Does not use all personal protective equipment (PPE)	Safety training for workers

## Appendix A

There are 40 unsafe behaviors occurring in all types of accidents, but 37 in the four types of accidents. The 40 unsafe behaviors and their nodes are shown in Table A1 as follows:

**Table A1:** The list of 40 unsafe behaviors involved in all accident cases.

Unsafe Behavior Node	Unsafe Behaviors
*B*_1_	Stay in unsafe areas
*B*_2_	Enter into dangerous areas
*B*_3_	Use overloaded machines
*B*_4_	Start/shut down/move machines and equipment without permissions
*B*_5_	Forget to turn off machines and equipment
*B*_6_	Operate machines and equipment against the procedures
*B*_7_	Operate errors (refers to the operations of buttons, valves, wrenches, handles, etc.)
*B*_8_	Engage in clean up when machines and equipment are still running
*B*_9_	Use machines and equipment without conforming safety before
*B*_10_	Use machines and equipment without effective safety devices
*B*_11_	Implement inadequate protection measures for holes or borders
*B*_12_	Does not use all personal protective equipment (PPE)
*B*_13_	Misuse PPE
*B*_14_	Engage in specialized operation without a permit
*B*_15_	Store objects improperly
*B*_16_	Install or dismantle scaffolding against procedures
*B*_17_	Install or dismantle formwork support system against procedures
*B*_18_	Install or dismantle machines and equipment against procedures
*B*_19_	Lay steel bars against procedures
*B*_20_	Do not fasten workpieces tightly
*B*_21_	Violate operation orders
*B*_22_	Concreting against procedures
*B*_23_	Reinforce support measures ineffectively
*B*_24_	Do not set up safety nets as required
*B*_25_	Build work platforms against specifications
*B*_26_	Adjust errors causing ineffective safety devices
*B*_27_	Issue improper commands
*B*_28_	Operate cranes with improper methods
*B*_29_	Use ladders incorrectly
*B*_30_	Lift within power lines at less than the safe distance, and fail to take protective measures
*B*_31_	Do not hang warning signs in dangerous areas
*B*_32_	Place foundation pit slopes too steep and fail to take protective measures
*B*_33_	Enter into dangerous areas of lifting
*B*_34_	Use overloaded construction platforms/scaffolding/support systems/templates
*B*_35_	Set drainages and impermeable layers against procedures
*B*_36_	Work after drinking
*B*_37_	Carryout hazardous tasks without professional supervisions
*B*_38_	Do not timely and effectively deal with environmental problems (such as surrounding buildings, surrounding grounds)
*B*_39_	Set circuit breakers/earth leakage protective devices/short-circuit protections against procedures
*B*_40_	Do not replace the damaged and aged electrical equipment in a timely manner
